# Comparison of gridded precipitation datasets for rainfall-runoff and inundation modeling in the Mekong River Basin

**DOI:** 10.1371/journal.pone.0226814

**Published:** 2020-01-08

**Authors:** Sophal Try, Shigenobu Tanaka, Kenji Tanaka, Takahiro Sayama, Chantha Oeurng, Sovannara Uk, Kaoru Takara, Maochuan Hu, Dawei Han

**Affiliations:** 1 Graduate School of Engineering, Kyoto University, Kyoto, Japan; 2 Faculty of Hydrology and Water Resources Engineering, Institute of Technology of Cambodia, Phnom Penh, Cambodia; 3 Disaster Prevention Research Institute, Kyoto University, Kyoto, Japan; 4 Department of Civil and Environmental Engineering, Tokyo Institute of Technology, Tokyo, Japan; 5 Graduate School of Advanced Integrated Studies in Human Survivability, Kyoto University, Kyoto, Japan; 6 Department of Civil Engineering, University of Bristol, BS8 1TR, Bristol, United Kingdom; Bristol University/Remote Sensing Solutions Inc., UNITED STATES

## Abstract

Precipitation, as a primary hydrological variable in the water cycle plays an important role in hydrological modeling. The reliability of hydrological modeling is highly related to the quality of precipitation data. Accurate long-term gauged precipitation in the Mekong River Basin, however, is limited. Therefore, the main objective of this study is to assess the performances of various gridded precipitation datasets in rainfall-runoff and flood-inundation modeling of the whole basin. Firstly, the performance of the Rainfall-Runoff-Inundation (RRI) model in this basin was evaluated using the gauged rainfall. The calibration (2000–2003) and validation (2004–2007) results indicated that the RRI model had acceptable performance in the Mekong River Basin. In addition, five gridded precipitation datasets including APHRODITE, GPCC, PERSIANN-CDR, GSMaP (RNL), and TRMM (3B42V7) from 2000 to 2007 were applied as the input to the calibrated model. The results of the simulated river discharge indicated that TRMM, GPCC, and APHRODITE performed better than other datasets. The statistical index of the annual maximum inundated area indicated similar conclusions. Thus, APHRODITE, TRMM, and GPCC precipitation datasets were considered suitable for rainfall-runoff and flood inundation modeling in the Mekong River Basin. This study provides useful guidance for the application of gridded precipitation in hydrological modeling in the Mekong River basin.

## Introduction

Annual flooding is an important hydrological characteristic of the Mekong River Basin (MRB), especially in the lower Mekong River in which flooding is a way of life. One the one hand, prolonged floods challenge the survival and sustainability of the local community, causing huge socio-economic damages. The annual average cost of the flood damages in the Lower Mekong Basin (LMB) ranges between 60 and 70 million US$ [1, 2]. The flood in 2011 caused more than 430 million US$ and the death toll reached 396 [3]. On the other hand, flooding drives the high productivity of the ecosystem and biodiversity in the downstream floodplains [4, 5]. It is critically important to understand the characteristics of the hydrological regime in the MRB for sustainable development and flood management.

Hydrological modeling is an effective approach to extrapolate and interpolate missing information over time and space between observations for hydrological assessment [6]. Oeurng et al. [7] studied Tonle Sap sub-basin of MRB using the SWAT model. Try et al. [8] applied the RRI model for a single flood event in the LMB. Tanaka et al. [9] investigated the flood characteristics in the Tonle Sap floodplain using an integrated hydrological-hydraulic model. However, the study of the hydrological regime over the whole MRB using a reliable model and related input is still lacking and needs to be fully addressed.

Precipitation is useful for understanding the mechanism of hydrological system and is the most important input data in the hydrological and hydraulic modeling [10]. Therefore, accurate precipitation data is required for effective hydrological studies. The available ground precipitation data at country level in the MRB is limited [11]. Thus, it is necessary to evaluate and use gridded rainfall products which are widely available. To date, the evaluation of gridded precipitation has been conducted in several sub-basins of the MRB [12–14]. However, the performance evaluation of gridded precipitation for flood-inundation modeling in the whole MRB has not been reported yet. Therefore, this study aims 1) to evaluate the performance of a rainfall-runoff-inundation model in the whole MRB for river discharge and flood inundation prediction; 2) to assess the performances of different gridded precipitation datasets in simulating the river discharge in the whole MRB and flood inundation in the LMB.

## Methodology

### Study area

**Fig 1:**
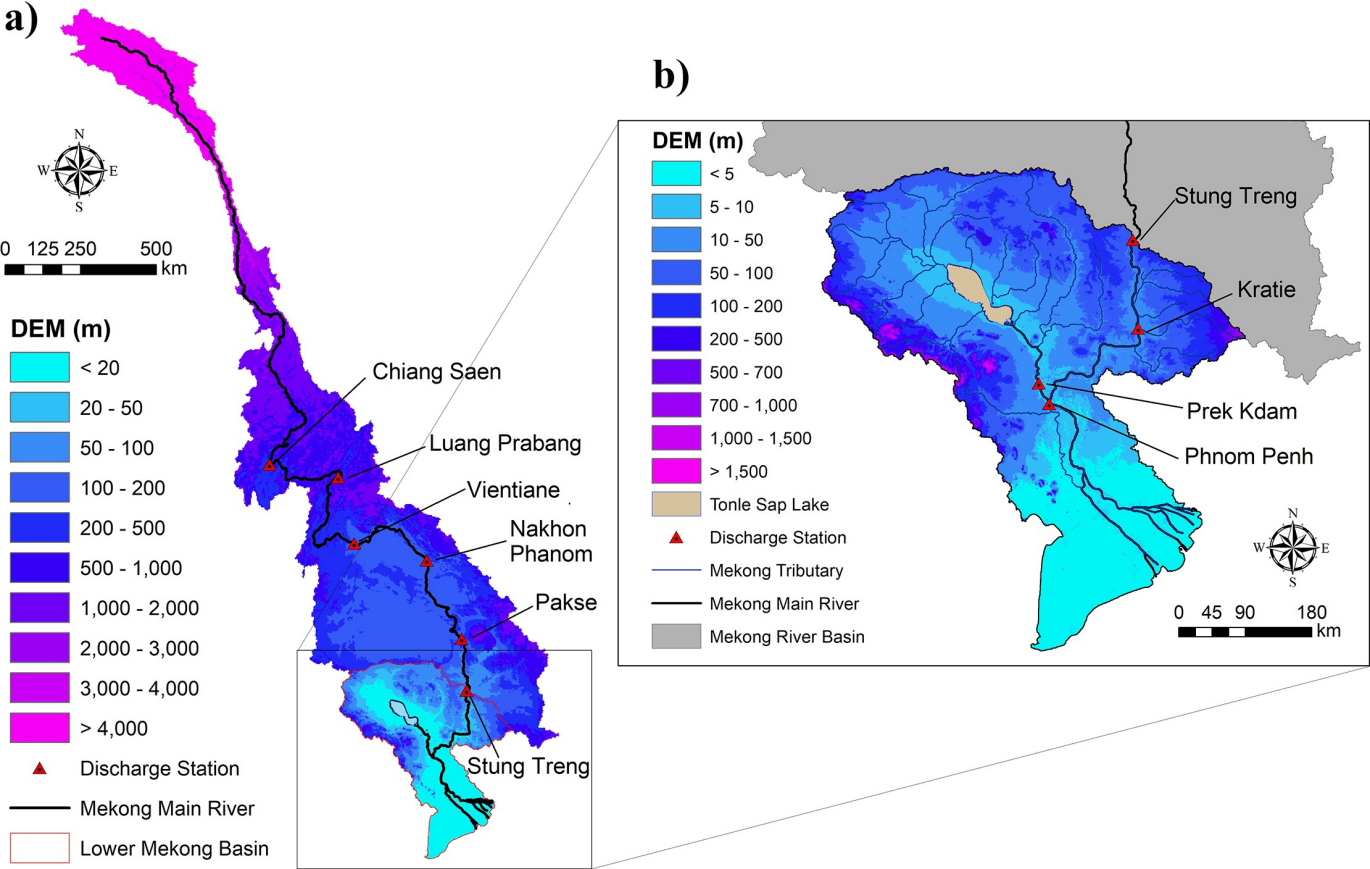
Location of the Mekong River Basin (a) and the Lower Mekong Basin (b).

The Mekong River is one of the longest river networks in the world, flowing through China, Lao PDR, Myanmar, Thailand, Cambodia, and Vietnam. It covers a vast area of 795,000km^2^ and supports a population of approximately 70 million people. The average discharge of the Mekong River is approximately 14,500m^3^/s (475km^3^/year) [1]. This study has assessed the hydrological process in the whole MRB and focused mainly on flood inundation in the downstream region where Cambodia’s Tonle Sap Lake and Vietnam’s Mekong delta are located (Fig 1). The Tonle Sap Lake is one of the most important natural resources in Asia that supports the people living inside and the surrounding areas of its floodplain [15–17]. The annual hydrological regimes in the MRB had a strong seasonal change. The Tonle Sap River connects Phnom Penh, the intersection of the Mekong’s mainstream, the Tonle Sap River, and the Bassac River, to the Tonle Sap Lake. In the wet season, water flows from the upstream of the Mekong River into the Tonle Sap Lake, and the flow reverses its course in the dry season to discharge water from the lake back into downstream of the Mekong, which finally reaches the Mekong delta.

### Rainfall-Runoff-Inundation model

This study used the Rainfall-Runoff-Inundation (RRI) model which is a 2D distributed model capable of simulating rainfall-runoff and flood inundation simultaneously [18]. The model has been applied in various basins to simulate large-scale flooding, to conduct hazard mapping and real-time inundation prediction. It is also used to elucidate flooding characteristics as well as to assess flood risk at a river basin scale [19–23].

At the stream network cell level, the model assumes that both river channel and surrounding slopes are located in the same grid. The model slope grid cells receive rainfall and flow based on the 2D diffusive wave equations, while the in-channel flow is calculated with the 1D diffusive equations. The RRI model simulation deals with surface and subsurface flow in the mountainous area and the Green-Ampt infiltration method in the floodplain separately. The flow interaction between the river channel and the slope is computed at a running time step interval based on different overflowing formulae, which depends on water-level and levee-height conditions. The RRI model provides the output of river discharge, river water level, inundation area and depth at the same time. The model was integrated into the global optimization algorithm of the Shuffled Complex Evolution (SCE-UA) tool [24] for calibration of its sensitive parameters. This model was applied with a spatial resolution of 2.5 arc-minute to understand the hydrological system for the whole MRB (Fig 1A) and 1.5 arc-minute for the LMB (Fig 1B) for more accurate inundation estimation. The time series of the river discharge at Stung Treng station was used as the boundary condition during the LMB simulation.

### Input data

The topography data including digital elevation model (DEM), flow direction (DIR), and flow accumulation (ACC) were obtained from the Multi-Error-Removed-Improved-Terrain (MERIT DEM) at the original resolution of 3-arc second (approx. 90 m at the equator) [25]. A topographic data scale-up algorithm in the RRI model was applied to transform the topography data to 1.5 and 2.5 arc minutes for LMB and MRB respectively. The land use data was obtained from the MODIS Land Cover Type Product (MCD12Q1) [26]. The surface evaporation was from the Japanese 55-year Reanalysis dataset (JRA-55) with a spatial resolution of 0.5625° and 3-hour temporal resolution [27].

### Precipitation datasets

This study used five gridded precipitation datasets including APHRODITE, GPCC, PERSIANN-CDR, GSMaP-RNL, and TRMM-3B42V7. Those datasets were chosen as a wide range of precipitation datasets at different spatial and temporal resolutions should be explored for an informative assessment. Brief information on the gridded rainfall products used in this study is illustrated in Table 1. The basin average annual precipitation recorded by the rain gauge is 1,488 mm/year, APHRODITE 1,349 mm/year, GPCC 1,588 mm/year, PERSIANN-CDR 1,720 mm/year, GSMaP-RNL 1,145 mm/year, and TRMM-3B42V7 1,393 mm/year.

**Table 1:** Description of the gridded precipitation datasets used in this study.

Dataset	Version	Spatial/temporal resolution	Area coverage	Period
APHRODITE [28]	V1801R1	0.25°/daily	Monsoon Asia	1998–2015
GPCC [30]	V.2018 (V2)	1°/daily	Global	1982–2016
PERSIANN [31]	CDR	0.25°/daily	Near global	1983-present
GSMaP [33]	RNL	0.1°/hourly	Near global	2000-present
TRMM [32]	3B42V7	0.25°/3-hourly	Near global	1998-present

#### APHRODITE dataset

The APHRODITE rainfall product is created by collecting and analyzing data from the gauged rainfall from 5,000–12,000 stations across Asia [28]. This product was produced by a joint project from the Research Institute for Humanity and Nature and Meteorological Research Institute covering 1951 to 2007 for Version V1101 and 1998–2015 for Version V1801R1. This study used APHRODITE Version V1801R1 with the daily temporal resolution and the spatial resolution of 0.25° [29].

#### GPCC dataset

The Global Precipitation Climatology Center (GPCC) Full Data Daily Version 2018 is based on the gauged precipitation from 67,200 stations worldwide provided by national meteorological and hydrological services, regional and global data collection organizations such as the World Meteorological Organization [30]. This product contains daily precipitation from 1982–2016 with the spatial resolution of 1° covering latitude: -90° to 90° and longitude: -180° to 180°.

#### PERSIANN dataset

Precipitation Estimation from Remotely Sensed Information using Artificial Neural Networks–Climate Data Record (PERSIANN-CDR) is developed by the Center for Hydrometeorology and Remote Sensing measures rainfall using infrared (IR) brightness temperature data from geostationary satellites [31]. PERSIANN is daily and 0.25° in space covering 60°S to 60°N from 1983 to the present.

#### TRMM dataset

The Tropical Rainfall Measuring Mission (TRMM) Multisatellite Precipitation Analysis (TMPA) is a product resulting from the combination of precipitation from multiple satellites and raingauges [32]. The data covers the latitude from 50°S to 50°N from 1998 to the present. TRMM 3B42 algorithm version 7 (TRMM-3B42V7) at fine spatial and temporal scales (0.25°×0.25° and 3-hourly) was used in this study.

#### GSMaP dataset

The Global Satellite Mapping of Precipitation (GSMaP) is derived from Precipitation Radar (PR), statistical classification, and scattering algorithms [33]. This study used GSMaP reanalysis version (GSMaP-RNL) precipitation available hourly from 2000 to date with a fine resolution of 0.1° covering 60°S to 60°N.

### Evaluation approach of gridded precipitation datasets

The present study focused on the period from 2000 to 2007, due to the existence of the largest number of rainfall gauged stations and few missing data during this period. The RRI model was calibrated and validated using the gauged rainfall during 2000–2007 as the gauged data from 2000 to 2007 used in this study showed good quality and density. Gauged rainfall has been commonly used for hydrological model calibration [12, 34]. Meanwhile, it is reported that model calibration using gridded data would produce unrealistic parameters [14, 35]. The calibrated model were used to simulate river flow and flood inundation using the gridded precipitation datasets. To evaluate the performance of streamflow simulation, we used three indicators including Nash-Sutcliffe model efficiency (NSE), coefficient of determination (R^2^), and relative volume error (VE), as follows:

$$\mathrm{NSE}=1-\frac{\sum {\left(Q_{sim}(t)-Q_{obs}(t)\right)}^{2}}{\sum {\left(Q_{obs}(t)-\bar{Q_{obs}}\right)}^{2}}$$

$$R^{2}=\frac{\sum {\left((Q_{sim}(t)-\bar{Q_{sim}}){(Q}_{obs}(t)-\bar{Q_{obs}})\right)}^{2}}{\sum {\left(Q_{sim}(t)-\bar{Q_{sim}}\right)}^{2}\sum {\left(Q_{obs}(t)-\bar{Q_{obs}}\right)}^{2}}$$

$$\mathrm{VE}=\frac{\sum Q_{sim}(t)-\sum Q_{obs}(t)}{\sum Q_{obs}(t)}$$

where *Q_sim_*(*t*) and *Q_obs_*(*t*) are the simulated and observed discharges at time step *t*, and $\bar{Q_{sim}}$ and $\bar{Q_{obs}}$ are the simulated and observed average discharges.

To evaluate the performance of inundation simulation, we used three indices including true ratio (TR), hit ratio (HR), and normalized error (NE), as follows:

$$\mathrm{TR}=\frac{{\mathrm{IC}}_{\mathrm{obs}}{\cap \mathrm{IC}}_{\mathrm{sim}}}{{\mathrm{IC}}_{\mathrm{sim}}}$$

$$\mathrm{HR}=\frac{{\mathrm{IC}}_{\mathrm{obs}}{\cap \mathrm{IC}}_{\mathrm{sim}}}{{\mathrm{IA}}_{\mathrm{obs}}}$$

$$\mathrm{NE}=\frac{{\mathrm{IC}}_{\mathrm{sim}}{‐\mathrm{IC}}_{\mathrm{obs}}}{{\mathrm{IC}}_{\mathrm{obs}}}$$

where IC_sim_ and IC_obs_ are the number of inundated cells from the model simulation and MODIS observation data.

## Result and discussion

### RRI model calibration and validation

#### River discharge

The model calibration and validation were carried out using the raingauge precipitation between 2000–2003 and 2004–2007, respectively. The RRI model was calibrated using an automatic global optimization algorithm called the Shuffle Complex Evolution (SCE-UA) at Stung Treng to evaluate the characteristics of the upstream area. Then, the daily discharge at this station was extracted as the input for the boundary condition to simulate the flood inundation performance of the downstream of the Mekong River.

**Fig 2:**
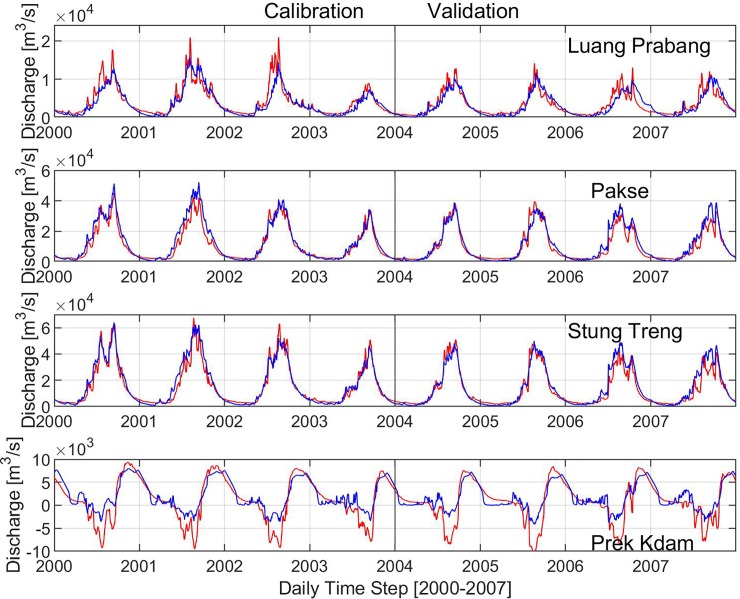
Simulated (blue) and observed (red) discharge during the calibration and validation periods at Luang Prabang (a), Pakse (b), Stung Treng (c), and Prek Kdam (d). (Note: The positive value at Prek Kdam represents the flow from Phnom Penh to the Tonle Sap Great Lake; the negative value indicates the reversed flow from the Tonle Sap lake to Phnom Penh.).

The comparison of the simulated and observed river discharges in Luang Prabang, Parkse, Stung Treng, and Prek Kdam is shown in Fig 2. It was found that there is a good agreement between the observation and simulation. The Stung Treng station provided the highest statistical performance indices with NSE = 0.94, R^2^ = 0.94, VE = 0.05 in the calibration and NSE = 0.89, R^2^ = 0.92, VE = 0.09 in the validation (Table 2). The statistics at Pakse were NSE = 0.90 and 0.89, R^2^ = 0.94 and 0.91, VE = 0.14 and 0.09 during calibration and validation, respectively. For tmost upstream located areas at Luang Prabang, the evaluation indicators were NSE = 0.81, R^2^ = 0.83 and -0.13 in the calibration and NSE = 0.77, R^2^ = 0.78, VE = 0.06 in the validation. NSE and R^2^ at Prek Kdam were 0.75 and 0.84 in the calibration and 0.69 and 0.77 in the validation. The coefficients of relative volume error VE = -0.27 and -0.31 in Prek Kdam were calculated by the assumption of the absolute value of inflow and outflow. This underestimated prediction might be due to the uncertainty of the topographic information.

**Table 2:** Model performance of the river discharge evaluation at the gauging stations during calibration (2000–2003) and validation (2004–2007) periods.

Dataset		NSE	R^2^	VE
Luang Prabang	Calibration	0.81	0.83	-0.13
	Validation	0.77	0.78	-0.06
Pakse	Calibration	0.90	0.94	0.14
	Validation	0.89	0.91	0.09
Stung Treng	Calibration	0.94	0.94	0.05
	Validation	0.89	0.92	0.09
Prek Kdam	Calibration	0.75	0.84	-0.27
	Validation	0.69	0.77	-0.31

#### Flood inundation

**Fig 3:**
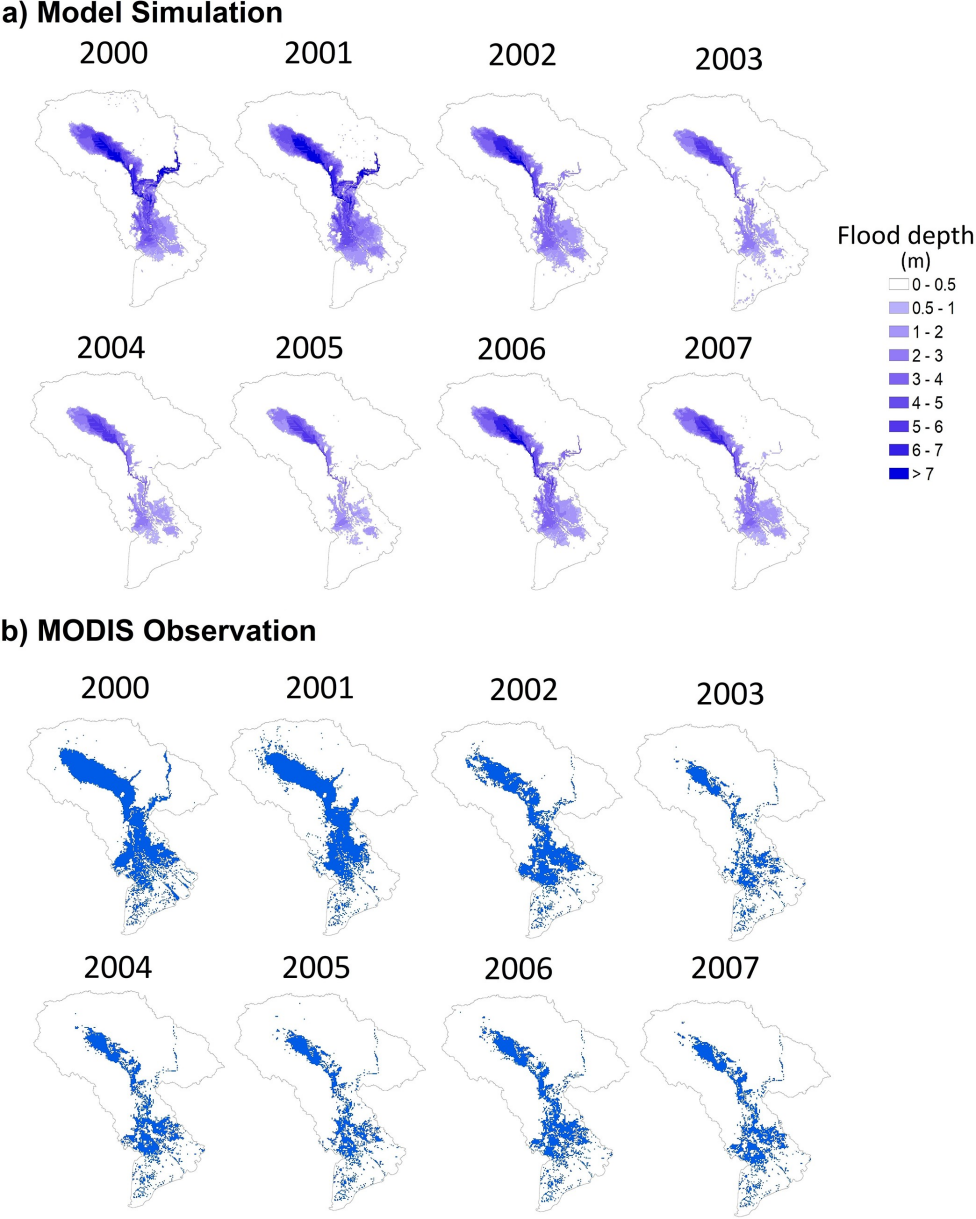
Model simulation (upper) and MODIS flood observation (lower) of the annual maximum flood extent from 2000 to 2007.

For inundation estimation, the annual peak flood extent in the LMB during 2000–2007 was compared with the MODIS flood observation dataset (Fig 3). This study selected the threshold value 0.5 m of water depth to distinguish between the flood and non-flood areas. Previous studies [21, 22, 36] have chosen this threshold value since water level is related to severe flood damage in the floodplain where the agricultural area is dominant land use type [37]. According to the performance indices of the spatial inundation extent in Table 3, the RRI model simulated the flood extent with a good agreement of 84% accuracy (i.e. the hit ratio in 2000 was 0.84). On the other hand, the hit ratio in 2006 was 0.89 corresponding to the accuracy of 89% of the simulated inundation area. The average values are TR = 0.68, HR = 0.81, and NE = 0.23; and TR = 0.56, HR = 0.80, and NE = 0.43 during calibration (2000–2003) and validation (2004–2007) respectively. the flood inundation simulation in this study was better than the previous study by Sayama et al. [22] in Chao Phraya River Basin in term of true ratio and hit ratio (i.e. their average values during 2005–2011 were TR = 0.41 and HR = 0.30). However, the normalized error value in the Chao Phraya case study was lower than that of this study (NE = -0.18).

**Table 3:** Model performance of the flood inundation extent compared with the remote sensing dataset.

	Year	IC_obs	IC_sim	IC_obs∩IC_sim	TR	HR	NE
Calibration	2000	5177	5011	4187	0.84	0.81	-0.03
	2001	4731	5610	3973	0.71	0.84	0.19
	2002	3860	4697	3257	0.69	0.84	0.22
	2003	2093	3280	1596	0.49	0.76	0.57
				Avg.	0.68	0.81	0.23
Validation	2004	2597	3307	1972	0.60	0.76	0.27
	2005	2204	2915	1590	0.55	0.72	0.32
	2006	2955	4562	2627	0.58	0.89	0.54
	2007	2526	3942	2125	0.54	0.84	0.56
				Avg.	0.56	0.80	0.43

### Performances of gridded precipitation datasets

**Fig 4:**
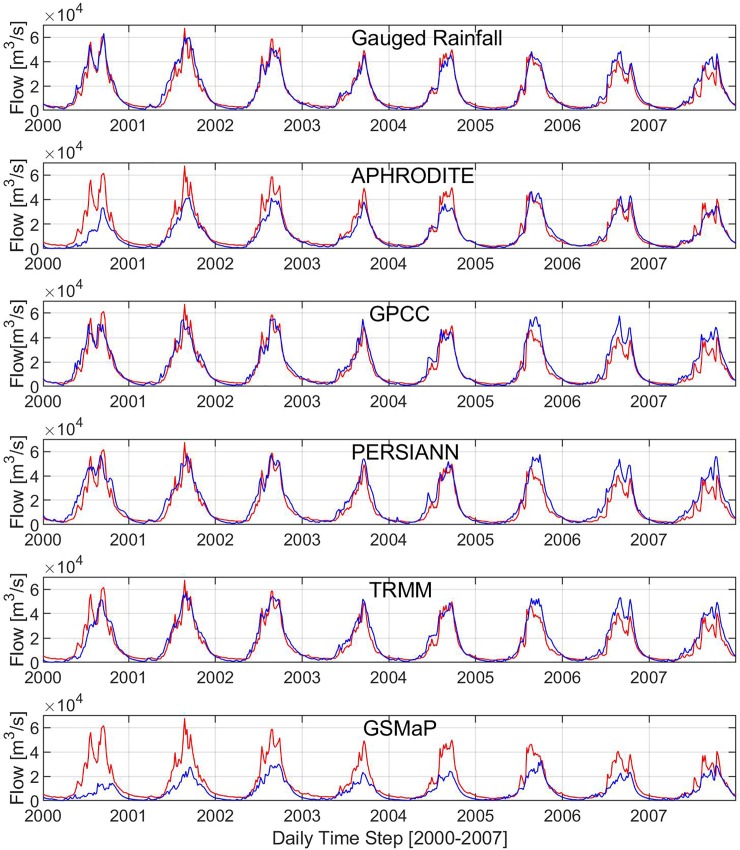
Observed (red) and simulated discharge (blue) from individual precipitation datasets at Stung Treng.

**Fig 5:**
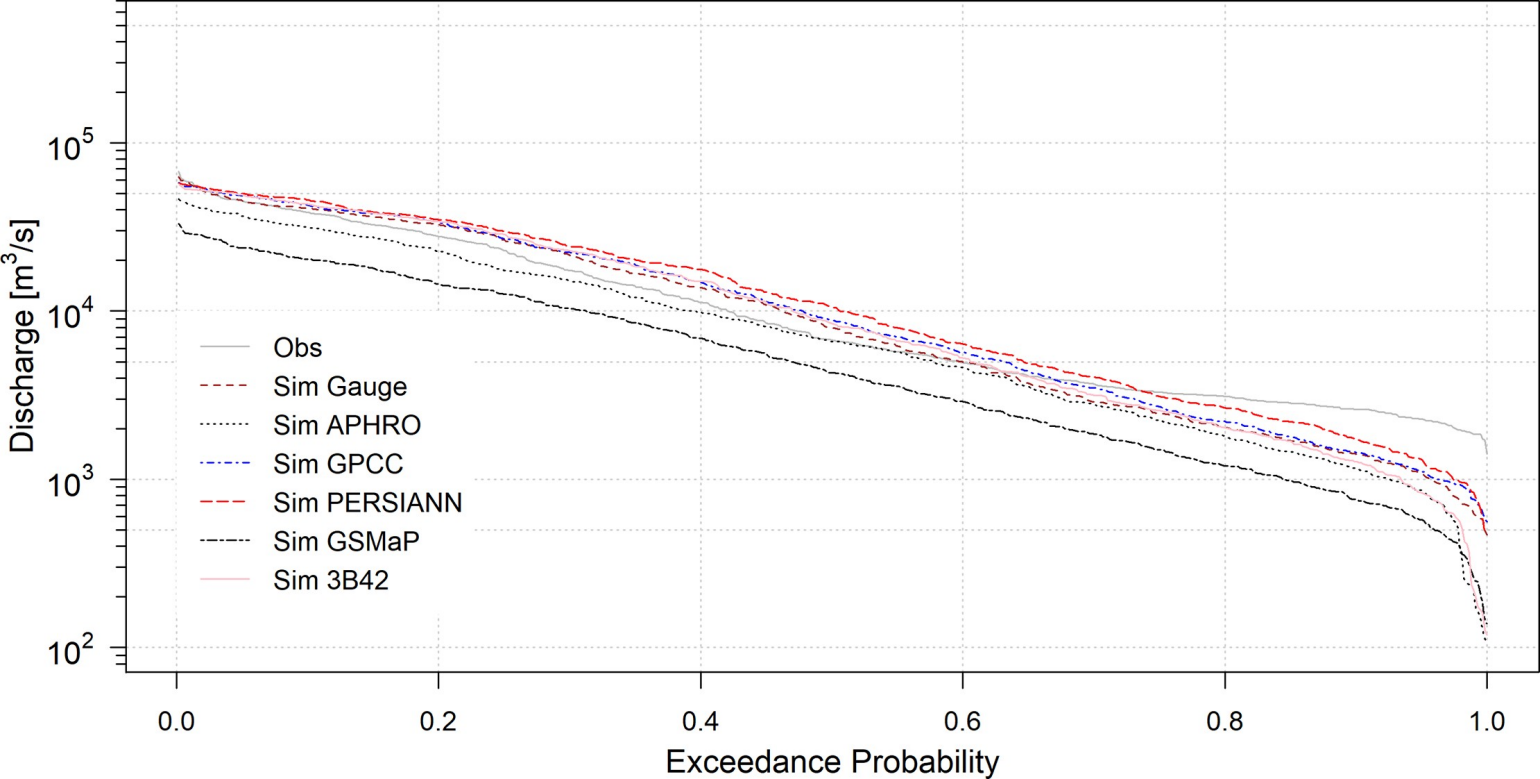
Flow duration curve of the simulated discharge.

After model calibration and validation, the same parameter setting was used to simulate river discharge and flood inundation using the five gridded precipitation datasets during 2000–2007. Fig 4 illustrated the observed and simulated discharge from all the precipitation datasets at Stung Treng. The performance indices include NSE from 0.42 to 0.92; R^2^ from 0.73 to 0.93; and VE from -0.46 to 0.21 (Table 4). The results of the river discharge indicated that APHRODITE, TRMM and GPCC datasets performed better with NSE = 0.81, 0.85, 0.84; R^2^ = 0.90, 0.89, 0.88; and VE = -0.19, 0.12, 0.13 at Stung Treng station followed by PERSIANN, and GSMaP. In addition, the extreme flow of the highest 5% of flow (Q_5_) from the flow duration curve was evaluated (Fig 5). The ratio of Q_5_ from the simulated discharges using the individual precipitation datasets were 1.00, 0.82, 1.09, 1.12, 0.53, 1.10 for rain-gauge, APHRODITE, GPCC, PERSIANN, GSMaP, and TRMM respectively.

**Table 4:** Performance indices for individual precipitation datasets.

Dataset	NSE	R^2^	VE
Rain gauge	0.92	0.93	0.07
APHRODITE	0.81	0.90	-0.19
GPCC	0.84	0.88	0.13
PERSIANN	0.80	0.88	0.21
GSMaP	0.42	0.73	-0.46
TRMM	0.85	0.89	0.12

The results of the average annual maximum flood extents in the simulation period (2000–2007) indicated that APHRODITE performed at the highest true ratio TR = 0.69 while the hit ratio indices of GPCC, PERSIANN, and TRMM were among the best (Table 5). The error indicators of APHRODITE and GSMaP were NE = -0.06 and 0.20 respectively followed by GPCC (NE = 0.58), TRMM (NE = 0.62), and PERSIANN (NE = 0.80).

**Table 5:** Statistical performance indices of the average annual maximum flood extents of the gridded precipitation datasets of 2000–2007.

Index	Gauge	APHRODITE	GPCC	PERSIANN	GSMaP	TRMM
TR	0.62	0.69	0.58	0.53	0.65	0.57
HR	0.81	0.62	0.86	0.91	0.69	0.86
NE	0.33	-0.06	0.58	0.80	0.20	0.62

GPCC was found to be the most suitable rainfall product to be used in hydrological modeling in the MRB followed by the APHRODITE and TRMM datasets. The GSMaP product underestimated the amount of rainfall while PERSIANN-CDR overestimated the rainfall in the MRB. This result agreed with the previous study by Try et al. [8] which validated and used the APHRODITE product for modeling a single flood event in the LMB. Guo et al. [38] found out that GSMaP and TRMM performed better while PERSIANN could not achieve good correlation coefficients in the Central Asia region. Tan et al. [39] mentioned that TRMM (3B42V7) and APHRODITE performed the best over Malaysia while PERSIANN-CDR had the worst performance. However, PERSIANN-CDR was found to underestimate the rainfall over the Luanhe River Basin, China, and the bias corrected version of TRMM (3B42) had the smallest error and highest correlation coefficient compard with the real-time version of TRMM (3B42RT) and PERSIANN-CDR.

Results from this study were in line with those of Thom et al. [14] indicating that the TRMM and APHRODITE datasets had good performances as input data to a hydrological model in the Srepok River Catchment, a tributary of the MRB. However, GPCC dataset were not evaluated in the above study [14]. Findings from the present study showed that the high resolution dataset did not always perform better in comparison with the coarse resolution datasets. For instance, GPCC at the coarsest resolution (1°) performed better than the other products while GSMaP (resolution 0.1°) did not perform well for a large scale basin such as the MRB. A similar conlusion was found by Vu et al. [40] where the GPCP rainfall product (i.e. resolution of 1°) was proved to be the second accurate dataset in the Dak Bla river basin, Vietnam.

## Conclusions

This study investigated the performance of the five gridded precipitation datasets in rainfall-runoff modeling and flood inundation simulation in the MRB. The results indicated that the RRI model performed well in the MRB. In addition, TRMM, GPCC, and APHRODITE had a better performance compared to GSMaP and PERSIANN-CDR for rainfall-runoff and inundation modeling in the whole MRB. GPCC and APHRODITE were found suitable for climate change studies and hydrological extreme event analysis in this region since these datasets provide long-term availability. Additionally, the TRMM dataset is available with 3-hour and daily temporal resolutions up to date, so it could be a useful data source for the flood event and real-time flood modeling. This study provides useful guidance for applications of the gridded precipitation for the hydrological modeling and assessing annual maximum inundated extents.
